# Role of the CHADS_2_ Score in the Evaluation of Carotid Atherosclerosis in Patients with Atrial Fibrillation Undergoing Carotid Artery Ultrasonography

**DOI:** 10.1155/2018/4074286

**Published:** 2018-08-19

**Authors:** Le-yu Lin, Lian-wei Yang, Yuan-yuan Shang, Yi-hui Li, Ming Zhong, Wei Zhang, Hui Zhu

**Affiliations:** ^1^The Key Laboratory of Cardiovascular Remodeling and Function Research, Chinese Ministry of Education and Chinese Ministry of Health and the State and Shandong Province Joint Key Laboratory of Translational Cardiovascular Medicine, Department of Cardiology, Qilu Hospital of Shandong University, Jinan, Shandong, China; ^2^Department of Emergency, Beijing Tiantan Hospital, Capital Medical University, Beijing, China; ^3^Department of Cardiology Group Two, Weihai Municipal Hospital, Weihai, Shandong, China; ^4^Department of Critical Care Medicine, Qilu Hospital of Shandong University, Jinan, Shandong, China

## Abstract

**Objective:**

This study investigated the characteristics of carotid atherosclerosis in patients with atrial fibrillation (AF) and determined the feasibility and significance of the CHADS_2_ score in predicting the degree of carotid atherosclerosis.

**Methods:**

Consecutive patients (n = 109) with nonvalvular AF were registered and classified into two groups, the paroxysmal AF group (n = 59) and persistent AF group (n = 50). Fifty healthy patients, matched by sex and age, were considered the control group. All patients were examined using carotid ultrasound and velocity vector imaging (VVI).

**Results:**

Compared with the control group, the mean intimal-medial thickness in the paroxysmal AF group (0.56 ± 0.11 versus 0.61 ± 0.10, respectively,* P *< 0.05) and the persistent AF group (0.56 ± 0.11 versus 0.64 ± 0.13, respectively,* P *< 0.001) was significantly increased. The plaque index (PI) in the persistent AF group was significantly higher than that observed in the paroxysmal AF group (1.05 ± 1.33 versus 1.42 ± 1.47, respectively,* P *< 0.001). Regarding the VVI indices, those reflecting the long-axis longitudinal motion function of carotid arteries were significantly decreased in both AF groups. Compared with the control group, a significantly lower total longitudinal displacement (tLoD) index was observed in the persistent AF group (0.73 ± 0.66 versus 0.31 ± 0.23, respectively,* P *< 0·0001) and the paroxysmal AF group (0.73 ± 0.66 versus 0.34 ± 0.17,* P *< 0·0001). The CHADS_2_ score was related to indicators reflecting the structure and function of the carotid artery.

**Conclusions:**

Carotid arterial structure and function were significantly altered in patients with AF. The degree of carotid atherosclerosis depended on the duration of AF. The CHADS_2_ score may be useful as a predictor of the extent of carotid atherosclerosis in patients with AF.

## 1. Introduction

Atrial fibrillation (AF) is one of the most common arrhythmias observed in clinical practice. Thromboembolic complications are the most serious consequences of AF [1]. Previous studies [2] documented that the main source of emboli in ischemic stroke with AF was left atrial thrombus. However, the major underlying causes of nonvalvular AF (hypertension, diabetes, coronary heart disease, and advanced age) have also been associated with a high risk for the development of atherosclerotic lesions. AF and atherosclerosis may mutually influence the development of each other [3, 4]. AF combined with carotid atherosclerotic stenosis may markedly increase the risk of recurrent ischemic stroke [5].

The CHADS_2_ (congestive heart failure, hypertension, age ≥75 years, diabetes mellitus, and prior stroke) score is used to evaluate the risk of stroke in patients with AF [6]. It has a strong predictive effect regarding the development of cardiac adverse events of left atrial origin in patients with AF [7, 8]. However, its role in predicting embolism of carotid plaque origin has not been determined.

Doppler ultrasound is able to evaluate the structure and function of the carotid artery intuitively to reflect related indices of atherosclerosis. Velocity vector imaging (VVI) is a novel ultrasound method, based on multiple M-mode evaluations and speckle-tracking technique [9, 10]. VVI allows the simultaneous evaluation of longitudinal and radial velocity, strain, strain rate, and displacement of the common carotid artery (CCA) wall motion [10]. The combination of VVI technology and Doppler ultrasound may evaluate the elasticity of the carotid vascular wall more comprehensively.

In the present study, Doppler ultrasound and VVI were used to explore the characteristics of carotid atherosclerosis in patients with AF and determine the feasibility and significance of the CHADS_2_ score in predicting the degree of carotid atherosclerosis.

## 2. Methods

### 2.1. Study Design

A total of 109 patients with nonvalvular AF fulfilling the inclusion criteria were enrolled in the study between September 2011 and May 2012. Of those, 59 patients had paroxysmal AF (22 males and 37 females, mean age: 58.32 ± 10.18 years [range: 30–75]) and the remaining 50 had persistent AF (18 males and 32 females, mean age: 59.30 ± 8.94 years [range: 25–78]). Nonvalvular AF was defined as AF in the absence of rheumatic mitral valve disease, a prosthetic heart valve, or mitral valve repair [11, 12]. AF which resolved spontaneously was designated as paroxysmal. Sustained AF which resolved through pharmacological intervention or electrical cardioversion was designated as persistent [12].

For the control group, 50 healthy patients (22 males and 28 females, mean age: 55.98 ± 7.19 years [range: 38–76]), matched by sex and age, were recruited from hospital staff and volunteers.

All patients were examined using electrocardiography (ECG) and 24-hour dynamic electrocardiography and echocardiography. Data including medical history, history of thromboembolism, history of drug use, and history of smoking/drinking were collected in detail.

### 2.2. Biochemical Assessments

All samples were collected following a 14-hour overnight fast and centrifuged within 30 minutes of collection. The levels of total serum cholesterol, low-density lipoprotein (LDL) cholesterol, triglyceride, high-density lipoprotein (HDL) cholesterol, and fasting blood glucose were analyzed from blood samples and measured using routine laboratory methods.

The study was conducted in accordance with the guidelines of the Declaration of Helsinki on biomedical research involving human subjects. Written informed consent was provided by all patients, and the study protocol was approved by the institutional ethics committee.

### 2.3. Acquisition of Carotid Ultrasound Index

The carotid ultrasound index of the study population was acquired using a Siemens Sequoia™ 512 color Doppler ultrasonic diagnostic apparatus. The probe frequency was 8.0–14.0 MHz. B-mode real-time ultrasound was performed to evaluate the wall thickness of the carotid artery. An expert sonographer, blinded to the data of the patients, performed the sonographic evaluations. Patients were examined in the supine position, as previously reported [13–15].

The probe was deployed approximately 1.5 cm proximally to the bifurcation of the CCA, and the longitudinal plane was used to visualize the maximum diameter of the lumen. Five segments were identified and measured in the anterior and posterior planes. At each of these sites the intimal-medial thickness (IMT) was determined, defined as the distance between the echogenic line representing the intimal blood interface and the outer echogenic line representing the adventitial junction. The maximum IMT value was determined as the mean IMT of the left and right arteries and was used for analysis.

During ultrasound assessments, the blood flow velocity in the carotid artery, arterial compliance, carotid stiffness index, resistance index, and other related indicators were measured. All detected plaques were counted, and their characteristics (fibrous, calcified or soft, presence, or absence of stenosis) were determined. The degree of plaque was graded on a scale from 0 to 3 (0 = no observable plaque; 1 = one small plaque [<30% of vessel diameter]; 2 = one medium plaque [30%–50% of vessel diameter] or multiple small plaques; and 3 = one large plaque [50% of vessel diameter] or multiple plaques with at least one medium plaque) [16].

### 2.4. Velocity Vector Imaging Acquisition and Analysis

All VVI-measurements were performed offline at a workstation by two independent observers. The VVI software (syngo® US workplace) was used to determine vessel wall velocity, strain, strain rate, and displacement of both the near and far wall of the CCA. Using the VVI mode of the device, the frame rate was adjusted to >60 frames/s. The transverse distal CCA approximately 1-cm inferior to the carotid bulb and free of plaque was used for analysis. Five guiding points were equally distributed (0.25-cm apart) within a 1-cm segment on the near wall and far walls; i.e., a total of 10 measuring points were used [17]. Dynamic images of three consecutive cardiac cycles were acquired and stored. The segmental values of the VVI parameters from both sides of the CCA were measured independently and averaged [17–19]. The total longitudinal displacement (tLoD) during a cardiac cycle was calculated as the sum of absolute values of the maximal systolic and maximal diastolic displacements. Patients with paroxysmal atrial fibrillation undergo carotid ultrasound during the onset of atrial fibrillation.

### 2.5. Statistical Analysis

All analyses were performed using the SPSS software (version 18.0, IBM Crop., Armonk, USA). Descriptive statistics were used to summarize the data. Categorical variables were expressed as percentages, while continuous variables were expressed as mean ± standard deviation. Differences between groups distributed variables were evaluated using one-way analysis of variance. Student's t-test was used to compare parameters between two groups. Correlation coefficients and their significance were calculated using Spearman's test. Possible predictors, identified through univariate analysis, were further analyzed using multiple logistic regression analyses to determine independent predictors. A p-value <0.05 denoted statistical significance in all analyses.

## 3. Results

### 3.1. Comparison of Clinical Characteristics between the AF and Control Groups

There were no statistically significant differences in sex and age among the three groups. In the paroxysmal AF and persistent AF groups, the proportion of patients with high systolic blood pressure, high waist-hip ratio, alcohol history, hypertension, diabetes, coronary heart disease, and hyperlipidemia was significantly increased compared with the control group (Table 1). In addition, the proportion of patients receiving concomitant treatment with antiplatelet therapy, anticoagulation therapy, angiotensin-converting enzyme inhibitors, *β*-receptor antagonists, and statins was significantly increased (Table 1). Of note, the proportion of patients suffering a stroke in the persistent AF group was significantly higher than that observed in the paroxysmal AF group (26% versus 5%, respectively,* P* < 0.01).

### 3.2. Comparison of Carotid Sonography Indices and VVI Indices between the AF and Control Groups (Grouped according to Type of AF)

Compared with the control group, in the paroxysmal AF group the mean IMT (0.56 ± 0.11 versus 0.61 ± 0.10, respectively,* P* < 0.05) and arterial compliance (AC) index (1.21 ± 1.23 versus 3.35 ± 2.57, respectively,* P* < 0.001) were significantly increased. The index for blood flow velocity in the carotid was significantly decreased (Table 2).

Compared with the control group, in the persistent AF group the mean IMT (0.56 ± 0.11 versus 0.64 ± 0.13, respectively.* P* < 0.001), AC index (1.21 ± 1.23 versus 3.14 ± 2.76, respectively,* P* < 0.05), and carotid stiffness index (*β*) (7.35 ± 5.95 versus 10.34 ± 9.32, respectively,* P *< 0.05) were significantly increased, whereas the index for the blood flow velocity in the carotid was significantly decreased (Table 2).

The plaque index (PI) was significantly higher in the persistent AF group than the paroxysmal AF group (1.05 ± 1.33 versus 1.42 ± 1.47, respectively,* P *< 0.001).

For the VVI indices, those reflecting the long-axis longitudinal motion and the long-axis radial motion of the carotid artery were significantly decreased in both AF groups compared with the control group (Table 2). The persistent AF group showed significantly lower tLoD compared with the control group (0.31 ± 0.23 versus 0.73 ± 0.66, respectively,* P *< 0.0001). This significant decrease in the tLoD index was also observed in the paroxysmal AF group (0.34 ± 0.17 versus 0.73 ± 0.66, respectively,* P *< 0.0001), suggesting that the long-axis motor function of the carotid artery was impaired in patients with AF.

**Figure 1:**
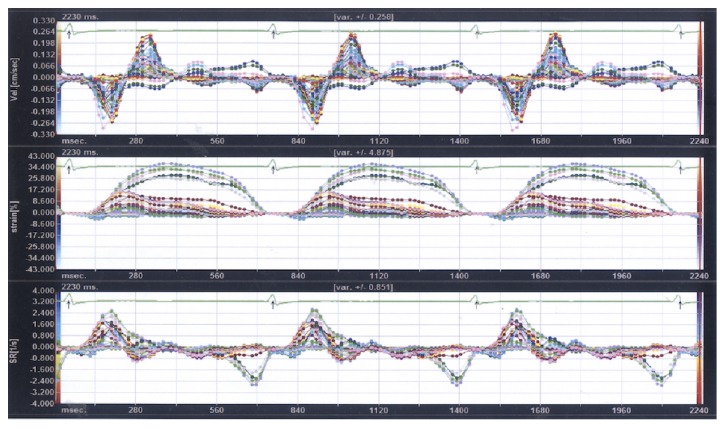
**Tracing curve of common carotid artery longitudinal motor function using velocity vector imaging in a patient with sinus rhythm.** The curves show regular synchronized motion of the common carotid artery intima during the cardiac cycle, with consistent velocity direction and magnitude.

**Figure 2:**
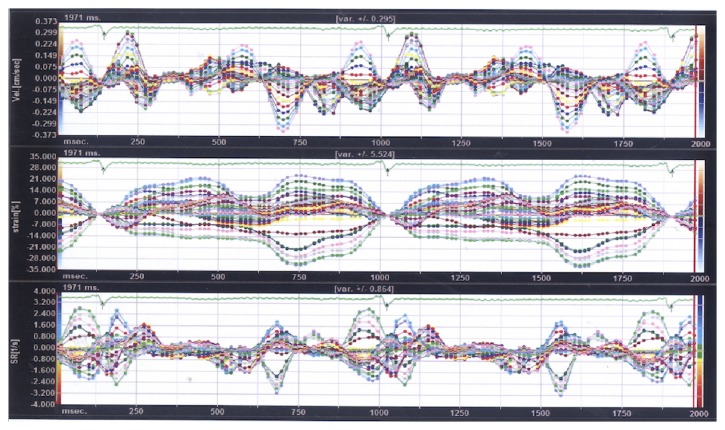
**Tracing curve of common carotid artery longitudinal motor function using velocity vector imaging in a patient with persistent atrial fibrillation.** The curves show that the motion of the common carotid artery intima at each point was desynchronized and the curves were disordered and irregular.

Detailed examples of output derived from the VVI software are shown in Figures 1 and 2. The Figures display longitudinal displacement curves during heart cycles in control subjects and patients with AF.

### 3.3. Comparison of Carotid Ultrasound Indices and VVI Indices between Patients (Grouped according to the CHADS_*2*_ Score)

Compared with the low-risk group (CHADS_2_ scores of 0), in the high-risk group (CHADS_2_ scores >2) the mean IMT (0.60 ± 0.12 versus 0.67 ± 0.10, respectively,* P *< 0.05), maximum IMT (0.75 ± 0.14 versus 0.86 ± 0.14, respectively,* P *< 0.01), and AC index (2.19 ± 0.95 versus 3.80 ± 3.19, respectively,* P *< 0.05) were significantly increased (Table 3). However, the index for blood flow velocity was significantly decreased (Table 3). The PI showed a tendency to increase, without reaching statistical significance. There were no statistically significant differences in these indices between the moderate-risk and high-risk groups.

### 3.4. Correlation Analysis between the CHADS_*2*_ Score, Carotid Sonography, and VVI Indices in the AF Groups

In patients with AF, the CHADS_2_ score was linked to indicators reflecting the structure of the carotid artery, including PI (r = 0.297,* P* = 0.002), mean IMT (r = 0.272,* P* < 0.001), and maximum IMT (r = 0.337,* P* < 0.001). Moreover, it correlated with indices reflecting carotid function (Table 4). Correlation analysis using the VVI indices showed that the CHADS_2_ score was correlated with indicators reflecting the long-axis longitudinal motion of the carotid artery (Table 4).

### 3.5. Logistic Regression to Identify Risk Factors for IMT Thickening (Table 5)

The CHADS_2_ score, total cholesterol, and LDL cholesterol were entered into the multivariate logistic regression analysis. The results showed that individuals with a high CHADS_2_ score [odds ratio (OR): 1.676, 95% confidence interval (CI): 1.075–2.661,* P *= 0.023], increased total cholesterol (OR: 0.225, 95% CI: 0.085–0.596,* P *= 0.003), and increased LDL cholesterol (OR: 14.526, 95% CI: 3.329–63.385,* P *< 0.001) were susceptible to IMT thickening.

## 4. Discussion

Clinical evidence has suggested that atherosclerosis and AF may be correlated and mutually promoted [20–22]. Patients with concurrent AF and atherosclerosis have a higher mortality rate compared with those diagnosed with either AF or atherosclerosis [23]. Studies have shown that the risk of developing AF is higher in patients with carotid atherosclerosis [21–23]. Other studies have also shown that AF itself may accelerate the occurrence and development of coronary atherosclerosis and myocardial infarction [20, 22].

Atherosclerotic plaques develop mainly in sites of frequent hemodynamic changes such as arterial bifurcations, openings, or curving. This suggests that abnormalities in hemodynamics play a major role in the development of atherosclerosis. In 1997, Minamino et al. [24] proposed that the abnormality in hemodynamics caused by AF was in part responsible for endothelial dysfunction and that endothelial injury was the initiating factor for atherosclerosis. Pober and Cotran proposed the shear stress hypothesis [25], suggesting that laminar flow shear stress may selectively induce the blood vessel endothelium to express “protective factors of atherosclerosis.” However, in cases of abnormal blood flow shear stress, this expression of protective factors in vascular endothelial cells is decreased or ceased. Therefore, the function of antiatherosclerosis would be inaccessible. Endothelium injury is the basis for the occurrence of atherosclerotic plaques.

Carotid arteries are useful for the observation of systemic atherosclerosis. Therefore, in the present study, high-resolution color Doppler ultrasound was used to evaluate the vascular structure and function of carotid arteries in patients with AF. The results indicated significant changes in carotid artery structure for patients with AF and a significant increase in the incidence and deterioration of atherosclerosis. At the same time, several indicators of carotid hemodynamics were altered, indicating decreased elasticity and increased stiffness in patients with AF. The PIs of carotid arteries in patients with persistent AF were significantly greater than those in patients with paroxysmal AF. This finding indicated that the development of atherosclerosis is dependent on the duration of AF [26, 27]. In our study, the data demonstrate that the systolic blood pressure was higher in both paroxysmal and persistent AF groups than in the control group. Hypertension is an important risk factor for arterial stiffness [28] and also for atrial fibrillation [6]. Studies [29, 30] and animal models of genetic or experimental hypertension [31–33] suggest hypertension exerts a direct effect on the cardiovascular system, stimulates hyperplasia and hypertrophy of vascular smooth muscle cells and adventitial cell migration, and may therefore lead to notable pathophysiological consequences, such as interfering with vessel mechanical properties. Therefore, the increased carotid stiffness in patients with atrial fibrillation in our study was also influenced by hypertension. Hypertension aggravates the hemodynamic changes in patients with atrial fibrillation, thereby promoting the structural changes and further reducing the function of the carotid artery.

Furthermore, VVI was used to observe and compare motion characteristics of the long axis of the CCA in patients with AF and controls. The results demonstrated long-axis motor dysfunction of the CCA in patients with AF. During VVI, patients traced in the two-dimensional image are tracked automatically in real time. Changes and intervals of pixels on each successive frame of the image are tracked and compared, thus displaying the true motion speed and displacement of the tissue in a vector manner and showing the indicators in curves. In this way, quantitative analysis for structural mechanics of the patients is possible in multiple planes and at various phases [19, 34]. Svedlund et al. used VVI to evaluate the long-axis motion of the CCA and found that the anterior and posterior walls of CCA in controls showed similar motion tendency. In contrast, the motion of the long axis of the CCA in patients with coronary atherosclerotic heart disease was significantly weaker than that observed in controls [17]. Therefore, VVI may be used to visually observe characteristic variations in the structural mechanics of the vascular endothelium and evaluate the elasticity of vascular walls [18, 19, 34]. In this study, regular synchronous motion was observed in the cardiac cycle of tunica intima of the CCA in patients with sinus rhythm, and the direction and size of the velocity vector tended to be inhibited. Nonetheless, the movements of the common carotid intima in patients with AF were not synchronous, with disordered and irregular curves, along with significantly impaired longitudinal and radial motor functions. These findings reflect stiffness and poor elasticity of the CCA wall and deteriorated atherosclerosis in patients with AF. Regional hemodynamic disturbances caused by AF may be a driving factor for carotid atherosclerosis. The shear stress of such abnormal blood flow causes damage to the carotid endothelium, becoming the initiating factor for the formation of atheromatous plaques [24, 25, 27].

The correlation between the CHADS_2_ score and carotid artery ultrasound indicators was investigated to estimate the degree and risk for the development of carotid atherosclerosis. The results showed that the CHADS_2_ score was correlated with multiple ultrasound indicators reflecting the structure and function of the carotid artery. The degree of carotid atherosclerosis in patients with a high CHADS_2_ score (≥2) was significantly higher than that observed in patients with a low-risk score (0). Therefore, the CHADS_2_ score is able to also predict the degree of carotid atherosclerosis in patients with nonvalvular AF. The increase in the score is accompanied with impaired structure and dysfunction of the carotid artery. This assessment method is simple and feasible, prompting physicians to determine the need for administration of statins in patients with a high CHAD_2_ score. This approach may alleviate the progression of atherosclerosis and reduce the risk of stroke.

## 5. Conclusions

The carotid arterial structure and function in patients with AF were significantly changed, including increased arterial wall stiffness, decreased elasticity, and aggravated atherosclerosis. A positive correlation between the CHADS_2_ score and carotid ultrasound indicators was found, indicating that risk stratification of AF stroke may also reflect the degree of structural and functional impairment of the carotid artery. Consequently, this may contribute to the assessment of the degree of carotid atherosclerosis.

## Figures and Tables

**Table 1:** Baseline clinical characteristics.

		Atrial fibrillation (n = 109)	
	Control group (n = 50)	Paroxysmal AF group (n = 59)	Persistent AF group (n = 50)
Age (years)	55.98 ± 7.19	58.32 ± 10.18	59.30 ± 8.94
SBP (mmHg)	122.74 ± 13.34	135.19 ± 19.41^*∗∗∗*^	137.48 ± 18.37^*∗∗∗*^
DBP (mmHg)	81.1 ± 611.29	79.51 ± 13.81	83.90 ± 12.44
Heart rate min^ −1^	66.60 ± 11.72	71.68 ± 24.20	88.58 ± 20.99^*∗∗∗*†††^
Body mass index (kg/m^2^)	24.76 ± 4.53	26.18 ± 3.52	26.12 ± 3.67
Waist-hip ration	0.87 ± 0.06	0.89 ± 0.06^*∗*^	0.91 ± 0.05^*∗∗∗*^
Fasting glucose (mmol/L)	5.42 ± 0.79	5.67 ± 2.04	5.48 ± 1.06
Cholesterol(mmol/L)	5.02 ± 0.99	4.77 ± 0.95	4.79 ± 1.40
HDL cholesterol (mmol/L)	1.35 ± 0.27	1.29 ± 0.30	1.23 ± 0.30
LDL cholesterol (mmol/L)	2.65 ± 0.62	2.74 ± 0.72	2.76 ± 1.02
Triglycerides (mmol/L)	1.50 ± 0.91	1.50 ± 0.74	1.73 ± 1.19
Male (%)	22 (44%)	37 (62%)	32 (64%)
Smoking history (%)	17 (33%)	23 (39%)	23 (46%)^*∗*^
Drinking history (%)	15 (29%)	27 (46%)^*∗∗*^	27 (53%)^*∗∗*^
Hypertension (%)	8 (16%)	36 (62^%^)^*∗∗*^	29 (57%)
Diabetes mellitus (%)	24 (4%)	12 (20%)^*∗*^	8 (16%)
CAD (%)	0	21 (36%)^*∗∗*^	21 (41%)^*∗∗*^
Prior Stroke (%)	0	3 (5%)	13 (26%)^*∗∗*††^
Statins (%)	0	13 (22%)^*∗*^	13 (26%)^*∗∗*^
Beta-blockers (%)	1 (2%)	36 (61%)^*∗∗*^	32 (64%)^*∗∗*^
ACEI (%)	0	22 (37%)^*∗∗*^	12 (23%)^*∗∗*^
ARB (%)	0	14 (24%)^*∗∗*^	20 (39%)^*∗∗*^
Anticoagulants (%)	1 (2%)	24 (41%)^*∗∗*^	19 (37%)^*∗∗*^
Aspirin (%)	2 (4%)	35 (60%)^*∗∗*^	36 (71%)^*∗∗*^
**CHADS**_**2**_ ** score**			
0 (%)	38 (76%)	20 (33.9%)	17 (34%)
1 (%)	12 (24%)	30 (50.8%)	12 (24%)
2 (%)	0	8 (13.6%)	14 (28%)
3 (%)	0	0	7 (14%)
≥4 (%)	0	1 (1.7%)	0

***Note*.** Compared with the control group, ^*∗*^*P*< 0.05; ^*∗∗*^*P*< 0.01; ^*∗∗∗*^*P*< 0.001.

Compared with the paroxysmal atrial fibrillation group, ^†^*P *< 0.05;^ ††^*P *< 0.01; ^†††^*P *< 0.001.

SBP, systolic blood pressure; DBP, diastolic blood pressure; CAD, coronary atherosclerotic heart disease; HDL, high-density lipoprotein; LDL, low-density lipoprotein; ACEI, angiotensin-converting enzyme inhibitors; ARB, angiotensin receptor blockers.

**Table 2:** Comparison of carotid ultrasound indices and VVI indices between the control group and the AF groups (grouped according to the type of AF).

		Atrial fibrillation (n = 109)	
	Control group (n = 50)	Paroxysmal AF group (n = 59)	Persistent AF group (n = 50)
Plaque index	0.16 ± 0.62	1.05 ± 1.33^*∗∗∗*^	1.42 ± 1.47^*∗∗∗*†††^
Mean IMT (mm)	0.56 ± 0.11	0.61 ± 0.10^*∗*^	0.64 ± 0.13^*∗∗∗*^
Max IMT (mm)	0.71 ± 0.13	0.77 ± 0.12	0.83 ± 0.15
Ds (mm)	6.71 ± 0.80	7.42 ± 1.14^*∗∗*^	7.72 ± 1.63^*∗∗∗*^
Dd (mm)	6.24 ± 0.82	6.95 ± 1.04^*∗∗*^	7.22 ± 1.63^*∗∗∗*^
Ep (kPa)	751.32 ± 682.21	876.08 ± 410.27	1091.29 ± 828.84^*∗*^
Ep^*∗*^	9.22 ± 7.68	11.42 ± 5.87	13.58 ± 12.76^*∗*^
DC (mmHg-1×10^ −2^)	0.15 ± 0.09	0.14 ± 0.06	0.14 ± 0.11
AC (mm^2^/kPa)	1.21 ± 1.23	3.35 ± 2.57^*∗∗*^	3.14 ± 2.76^*∗*^
*β*	7.35 ± 5.95	8.42 ± 3.92	10.34 ± 9.32^*∗*^
Vs (cm/s)	0.56 ± 0.12	0.49 ± 0.15^*∗*^	0.45 ± 0.20^*∗∗*^
Vd (cm/s)	0.29 ± 0.07	0.24 ± 0.07^*∗∗*^	0.24 ± 0.10^*∗∗*^
Vm (cm/s)	0.42 ± 0.09	0.35 ± 0.14^*∗∗*^	0.37 ± 0.11^*∗*^
Long Pv S (cm/s)	0.60 ± 0.71	0.31 ± 0.18^*∗*^	0.24 ± 0.20^*∗∗*^
Long Pv D (cm/s)	0.32 ± 0.19	0.21 ± 0.12^*∗∗*^	0.19 ± 0.13^*∗∗*^
Long Ps S (%)	5.04 ± 3.63	3.28 ± 2.13^*∗*^	2.67 ± 2.78^*∗∗*^
Long Ps D (%)	8.74 ± 8.48	4.86 ± 2.45^*∗∗*^	4.10 ± 3.37^*∗∗*^
Long Psr S (1/s)	1.21 ± 1.23	0.67 ± 0.39^*∗*^	0.57 ± 0.49^*∗∗*^
Long Psr D (1/s)	0.65 ± 0.41	0.51 ± 0.28^*∗*^	0.43 ± 0.31^*∗∗*^
Long Pd S (mm)	0.27 ± 0.24	0.13 ± 0.08^*∗∗*^	0.13 ± 0.12^*∗∗*^
Long Pd D (mm)	0.47 ± 0.53	0.21 ± 0.12^*∗∗*^	0.17 ± 0.15^*∗∗*^
tLoD (mm)	0.73 ± 0.66	0.34 ± 0.17^*∗∗∗*^	0.31 ± 0.23^*∗∗∗*^
Ra Pv S (cm/s)	0.22 ± 0.17	0.19 ± 0.18	0.14 ± 0.14^*∗*^
Ra Pv D (cm/s)	0.15 ± 0.12	0.10 ± 0.05^*∗*^	0.12 ± 0.11
Ra Pd S (mm)	0.10 ± 0.11	0.07 ± 0.06	0.06 ± 0.06^*∗*^
Ra Pd D (mm	0.18 ± 0.20	0.12 ± 0.08	0.09 ± 0.08^*∗*^

**Note: **compared with the control group, ^*∗*^*P*< 0.05; ^*∗∗*^*P*< 0.01; ^*∗∗∗*^*P*<0.001.

Compared with the paroxysmal atrial fibrillation group, ^†^*P *< 0.05; ^††^*P *< 0.01; ^†††^*P *< 0.001.

IMT, intima media thickness; VVI, velocity vector imaging; AC, arterial compliance; Ds, systolic diameter; Dd, diastolic diameter; Ep, pressure strain elastic modulus; DC, distensibility coefficient; *β*, stiffness index; Vs, systolic peak velocity; Vd, diastolic peak velocity; Vm, mean velocity; Long Pv S, systolic longitudinal peak velocity; Long Pv D, diastolic longitudinal peak velocity; Long Ps S, systolic longitudinal peak strain; Long Ps D, diastolic longitudinal peak strain; Long Psr S, systolic longitudinal peak strain rate; Long Psr D, diastolic longitudinal peak strain rate; Long Pd S, systolic longitudinal peak displacement; Long Pd D, diastolic longitudinal peak displacement; Ra Pv S, systolic radial peak velocity; Ra Pv D, diastolic radial peak velocity; Ra Pd S, systolic radial peak displacement; Ra Pd D, diastolic radial peak displacement; tLoD, total longitudinal displacement.

**Table 3:** Comparison of carotid ultrasound indices and VVI indices between the AF groups (grouped according to the CHADS_2_ score).

	score = 0 (n = 38)	Score = 1 (n = 42)	score ≥2 (n = 29)
Plaque index	0.78±1.03	1.46±1.57	1.52±1.48
Mean IMT (mm)	0.60±0.12	0.62±0.10	0.67±0.10^*∗*^
Max IMT (mm)	0.75±0.14	0.80±0.12	0.86±0.14^*∗∗*^
Ds (cm)	0.72±0.17	0.77±0.11	0.77±0.11
Dd (cm)	0.68±0.17	0.72±0.10	0.71±0.10
Ep (kPa)	887.81±854.52	1027.38±441.45	1017.86±574.22
Ep^*∗*^	12.25±14.20	12.58±5.64	12.42±6.88
DC (mmHg-1×10^−2^	0.14±0.06	0.12±0.06	0.16±0.14
AC (mm2/kPa)	2.19±0.95	3.84±3.02^*∗*^	3.80±3.19^*∗*^
*β*	9.26±10.21	9.39±4.14	9.25±4.93
Vs (cm/s)	0.51±0.16	0.48±0.19	0.42±0.15^*∗*^
Vd (cm/s)	0.26±0.07	0.23±0.09	0.21±0.08^*∗*^
Vm (cm/s)	0.39±0.11	0.35±0.13	0.32±0.11^*∗*^
RI	0.48±0.07	0.51±0.11	0.48±0.12
PI	0.63±0.12	0.60±0.21	0.65±0.21
Long Pv S (cm/s)	0.25±0.17	0.31±0.21	0.25±0.19
Long Pv D (cm/s)	0.18±0.10	0.21±0.13	0.22±0.14
Long Ps S (%)	2.58±1.86	3.65±2.74	2.62±2.58
Long Ps D (%)	3.94±2.21	5.05±3.19	4.49±3.25
Long Psr S (1/s)	0.57±0.34	0.72±0.47	0.58±0.50
Long Psr D (1/s)	0.43±0.25	0.52±0.32	0.47±0.32
Long Pd S (mm)	0.10±0.09	0.14±0.09	0.16±0.13^*∗*^
Long Pd D (mm)	0.17±0.12	0.23±0.16	0.16±0.10
tLoD (mm)	0.27±0.17	0.38±0.21	0.32±0.20
Ra Pv S (cm/s)	0.17±0.16	0.19±0.19	0.13±0.12
Ra Pv D (cm/s)	0.10±0.08	0.10±0.06	0.13±0.12
Ra Pd S (mm)	0.05±0.04	0.07±0.06	0.07±0.06
Ra Pd D (mm)	0.11±0.09	0.11±0.08	0.10±0.08

**Note:** compared with the low-risk group (score = 0), ^*∗*^*P*< 0.05; ^*∗∗*^*P*< 0.01; ^*∗∗∗*^*P*< 0.001.

IMT, intima media thickness; VVI, velocity vector imaging; AC, arterial compliance; Ds, systolic diameter; Dd, diastolic diameter; Ep, pressure strain elastic modulus; DC, distensibility coefficient; *β*, stiffness index; Vs, systolic peak velocity; Vd, diastolic peak velocity; Vm, mean velocity; Long Pv S, systolic longitudinal peak velocity; Long Pv D, diastolic longitudinal peak velocity; Long Ps S, systolic longitudinal peak strain; Long Ps D, diastolic longitudinal peak strain; Long Psr S, systolic longitudinal peak strain rate; Long Psr D, diastolic longitudinal peak strain rate; Long Pd S, systolic longitudinal peak displacement; Long Pd D, diastolic longitudinal peak displacement; Ra Pv S, systolic radial peak velocity; Ra Pv D, diastolic radial peak velocity; Ra Pd S, systolic radial peak displacement; Ra Pd D, diastolic radial peak displacement; tLoD, total longitudinal displacement.

**Table 4:** Correlation analysis of CHADS_2_ score, carotid ultrasound, and VVI indices.

	CHADS_2_ score	
	r	* P*
Plaque index	0.297	0.002
Mean IMT (mm)	0.272	<0.001
Max IMT (mm)	0.337	<0.001
Ds (cm)	0.345	<0.001
Dd (cm)	0.346	<0.001
Ep (kPa)	0.334	0.001
Ep^*∗*^	0.278	0.001
DC (mmH^−1^×10^−2^)	−0.157	0.047
AC (mm2/kPa)	0.252	0.001
*β*	0.259	0.001
Vs (cm/s)	−0.315	<0.001
Vd (cm/s)	−0.342	<0.001
Vm (cm/s)	−0.288	0.003
Long Pv S (cm/s)	−0.196	0.013
Long Pv D (cm/s)	−0.102	0.203
Long Ps S (%)	−0.134	0.092
Long Ps D (%)	−0.166	0.037
Long Psr S (1/s)	−0.191	0.016
Long Psr D (1/s)	−0.100	0.210
Long Pd S (mm)	−0.065	0.416
Long Pd D (mm)	−0.215	0.007
tLoD (mm)	−0.19	0.017
Ra Pv S (cm/s)	−0.127	0.112
Ra Pv D (cm/s)	0.012	0.884
Ra Pd S (mm)	0.023	0.77
Ra Pd D (mm)	−0.134	0.093

IMT, intima media thickness; VVI, velocity vector imaging; AC, arterial compliance; Ds, systolic diameter; Dd, diastolic diameter; Ep, pressure strain elastic modulus; DC, distensibility coefficient; Vs, systolic peak velocity; Vd, diastolic peak velocity; Vm, mean velocity; Long Pv S, systolic longitudinal peak velocity; Long Pv D, diastolic longitudinal peak velocity; Long Ps S, systolic longitudinal peak strain; Long Ps D, diastolic longitudinal peak strain; Long Psr S, systolic longitudinal peak strain rate; Long Psr D, diastolic longitudinal peak strain rate; Long Pd S, systolic longitudinal peak displacement; Long Pd D, diastolic longitudinal peak displacement; Ra Pv S, systolic radial peak velocity; Ra Pv D, diastolic radial peak velocity; Ra Pd S, systolic radial peak displacement; Ra Pd D, diastolic radial peak displacement; tLoD, total longitudinal displacement.

**Table 5:** Logistic regression to identify risk factors for IMT thickening.

	*β*	*P*	OR	95% CI
CHADS_2_ score	0.516	0.023	1.676	1.075–2.661
Cholesterol (mmol/L)	−1.491	0.003	0.225	0.085–0.596
LDL cholesterol (mmol/L)	2.676	<0.001	14.526	3.329–63.385

## Data Availability

The data used to support the findings of this study are included within the article.
